# Complete Genome Sequence of Bacteriophage SEN8, a Temperate Phage Isolated from *Salmonella enterica* subsp. *salamae*

**DOI:** 10.1128/genomeA.00239-17

**Published:** 2017-05-11

**Authors:** Juraj Bosák, Lenka Mikalová, Darina Čejková, Jan Šmarda, David Šmajs

**Affiliations:** a Department of Biology, Faculty of Medicine, Masaryk University, Brno, Czech Republic; b Veterinary Research Institute, Brno, Czech Republic

## Abstract

A temperate phage, SEN8, having a broad activity against pathogenic *Salmonella* serovars, was isolated from *Salmonella enterica* subsp. *salamae* strain Sen8. The complete genome sequence of phage SEN8 was determined (35,203 bp) and showed relatedness to P2-like phages (*Salmonella* phages Fels-2 and RE-2010).

## GENOME ANNOUNCEMENT

Infections of *Salmonella* spp. are an important public health problem. While *S. enterica* subsp. *enterica* causes about 99% of human salmonelloses (1), the remaining sporadic infections are caused by *Salmonella* strains from subspecies *salamae*, *arizonae*, *diarizonae*, and *houtenae* (2). Although phages and prophages of *S. enterica* subsp. *enterica* have been intensively studied (3), there is limited information available on phages from other *Salmonella* subspecies. Recently, we showed that almost 40% of *Salmonella* “non-*enterica*” isolates produced phages (4); additionally, their activity was tested against common pathogenic *S. enterica* subsp. *enterica* serovars. The broadest activity was found in *S. enterica* subsp. *salamae* strain 244/03 II (Sen8), which inhibited almost 80% of the tested pathogenic isolates. Strain Sen8 originated from the stool of a patient living in the Czech Republic. The complete genome sequence of the phage SEN8 (family: *Myoviridae*), isolated from the *S. enterica* subsp. *salamae* strain Sen8, is presented in this article.

Phage particles were multiplied from a single plaque on the *S. enterica* subsp. *salamae *indicator 356/03 (Sen9) and isolated using a CsCl gradient. The phage suspension was lysed using the SDS/proteinase K method, and DNA was purified using the phenol-chloroform method (4). A Nextera XT DNA library preparation kit (Illumina, Inc.) was used for preparation of the DNA library, and sequencing was carried out on an Illumina MiSeq instrument, resulting in 1,214,756 paired-end reads (728,853.6 kb). Quality trimming and *de novo* assembly were performed using Trimmomatic and IDBA_UD software, respectively (5, 6), resulting in a complete genome sequence with a length of 35,203 bp, a G+C content of 51.94%, and an average coverage of 4,853× (1,058,791 reads). The assembly of the whole-genome sequence was verified using a *Hin*dIII-restriction profile of phage DNA and pulsed-field gel electrophoresis. The genome was annotated using the RAST fully automatic annotation service (7), followed by manual correction based on the BLAST algorithm (8). Annotation resulted in the identification of a *cos* site (ggcgtggcggggatacgag) and in the prediction of 49 genes in the phage genome. Based on BLASTn analysis, the genome sequence of the SEN8 phage shared the greatest similarity (i.e., 77% and 68%) with *Salmonella* phages Fels-2 (NC_010463) and RE-2010 (NC_019488), respectively, both of which were P2-like phages. Unlike most of the SEN8 genome, the region encoding tail fiber proteins was not related to the Fels-2 and RE-2010 phages. Tail fiber proteins determine phage–host interactions and are potentially responsible for the observed broad activity of SEN8 against pathogenic serovars of *S. enterica*.

### Accession number(s).

The complete genome sequence of phage SEN8 was deposited in GenBank under the accession number KT630647.2.
